# Poking the cell surface: mechanics, remodeling, and what force reveals

**DOI:** 10.1007/s12551-026-01473-4

**Published:** 2026-10-09

**Authors:** Bruno Pontes

**Affiliations:** Instituto de Ciências Biomédicas & Centro Nacional de Biologia Estrutural E Bioimagem (CENABIO), Universidade Federal Do Rio de Janeiro, Rio de Janeiro, RJ 21941-902 Brazil

**Keywords:** Cell surface mechanics, Membrane tension, Viscoelasticity, Rheology, Mechanobiology, Mechanomics, Membrane-cortex adhesion, Force-driven remodeling

## Abstract

What happens when you poke a living cell, applying a localized external force to its surface? The answer depends, in part, on how long you keep poking. The plasma membrane and the actomyosin cortex beneath it form a mechanically active interface that changes in disease and can itself be reshaped by force. This review explores how the cell surface responds to such force in two overlapping experimental regimes. Poke briefly, over seconds, and force becomes a readout, revealing biophysical parameters that report on cell state and serve as candidate single-cell markers of disease. Keep poking, over minutes, and force can remodel the surface, suggesting that it may act not only as a perturbation but also as an instructive signal. What emerges is a mechanical fingerprint whose potential for diagnosis, disease monitoring, and, eventually, controlled manipulation of cell surfaces is only beginning to be understood.

## Introduction

Every cell is surrounded by a surface that is simultaneously a structural boundary, a signaling platform, and a mechanical interface. The plasma membrane and the underlying actomyosin cortex form a composite material whose physical properties regulate how cells maintain shape, respond to their environment, divide, migrate, and communicate with neighboring cells (Kelkar et al. 2020; Sitarska and Diz-Munoz 2020). The membrane-cortex complex is continuously and actively remodeled in response to biochemical signals, adhesive interactions, and mechanical forces (Salbreux et al. 2012; Chugh and Paluch 2018). Applying a localized force to this interface and measuring how it responds is one of the most direct ways to study it.

This approach has shown that the membrane-cortex complex has measurable mechanical parameters, including membrane tension, bending rigidity, membrane-cortex attachment (MCA), and viscoelastic moduli; that these parameters change in disease, as illustrated most clearly by red blood cells (RBCs); and that sustained force can remodel the cell surface, raising the possibility of links to protrusions and intercellular connections such as tunneling nanotubes (TNTs). Each of these topics has been covered by previous reviews: membrane tension and MCA, including their roles in health and disease (Pontes et al. 2017a; Le Roux et al. 2019; Sitarska and Diz-Munoz 2020; Li et al. 2024); the structure and mechanics of the actin cortex (Chugh and Paluch 2018; Kelkar et al. 2020); cell surface biophysics and microrheology based on optical tweezers (OT) (Nussenzveig 2018; Catala-Castro et al. 2022; Vos et al. 2024); techniques for measuring cell mechanics (Wu et al. 2018); single-cell mechanical phenotyping (Urbanska and Guck 2024); RBC mechanics and deformability (Liang et al. 2025; Yang et al. 2026); and TNT biology and biogenesis (Zurzolo 2021; Palese et al. 2025; Brou and Zurzolo 2026; Dias et al. 2026).

What these reviews do not provide, and what the present review attempts, is an integration of these topics around the duration of a localized force applied to the cell surface, a variable that determines the role that force plays. Two dominant experimental regimes can be distinguished. In the first, force is a quantitative readout: brief, localized perturbations yield the parameters listed above, as well as membrane-cortex friction, all of which report on cell state. In the second, force is sustained and becomes a potentially instructive stimulus that can recruit actin and reorganize the membrane-cortex interface. For brevity, these are referred to here as the “seconds” and “minutes” regimes, but they are conceptual categories with substantial temporal overlap, not strict temporal boundaries (“Sustained force (minutes): remodeling of the cell surface” section). The central question is what poking a cell, briefly or persistently, reveals about its mechanical state, and how that state changes in disease or under sustained force. The final sections address the diagnostic potential of cell surface mechanics, the requirements for its clinical translation, and the open questions that define the frontier of this field. 

## Probing the cell surface under force

**Fig. 1:**
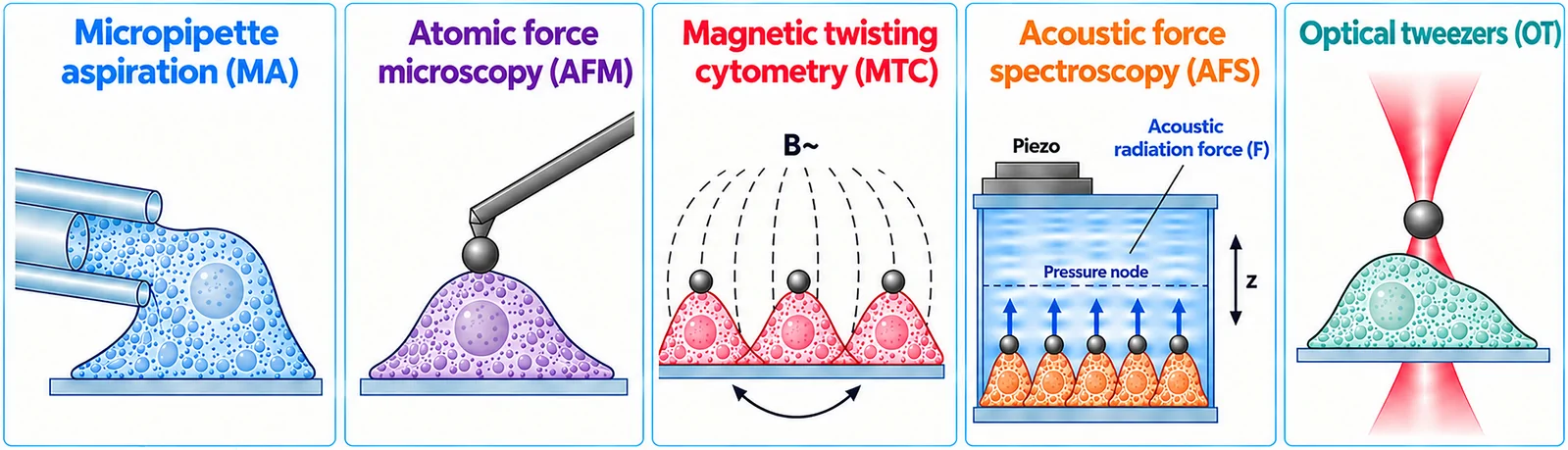
How each technique engages the cell surface. Schematic of five force-probing techniques. Micropipette aspiration (MA) applies a controlled suction pressure through a glass pipette. Atomic force microscopy (AFM) indents the surface with a cantilever tip. Magnetic twisting cytometry (MTC) applies a magnetic torque to ferromagnetic beads bound to cell surfaces. Acoustic force spectroscopy (AFS) uses a piezoelectric transducer to drive a bulk acoustic standing wave along the height (z) of a microfluidic chamber, and the resulting acoustic radiation force (F) acts on receptor-bound beads. Optical tweezers (OT) trap a micron-sized dielectric bead in a focused laser beam and use it as a force transducer at user-defined positions on the cell surface. Each technique occupies a different position in the trade-off between force range, spatial specificity, throughput and ease of simultaneous imaging. These characteristics, the practical determinants that shape what each technique actually measures, and the operational criteria used to compare them are summarized in Table 1

Probing the cell surface with localized forces requires tools that can apply and detect forces from nanonewtons down to sub-piconewtons (10^–9^ to 10^–12^ N), with spatial resolution from micrometers to nanometers (10^–6^ to 10^–9^ m), while the cell remains alive and, ideally, while the probed region can be imaged. Several such tools exist (Fig. 1; Table 1). Each is suited to a different question, and interpreting its results correctly requires knowing which question it answers (Wu et al. 2018).

**Table 1:** Comparison of the force-probing techniques discussed in the “Probing the cell surface under force” section, showing their measurement capabilities and the practical determinants of what each technique actually measures

Attribute	Micropipette aspiration (MA)	Atomic force microscopy (AFM)	Magnetic twisting cytometry (MTC)	Acoustic force spectroscopy (AFS)	Optical tweezers (OT)
Force range	Set by suction pressure (≈1 Pa to kPa); ≈pN–nN for pipette radii of a few µm	≈10 pN to > 100 nN	Magnetic torque, usually expressed as a specific torque (torque per bead volume, in Pa) rather than as a force per bead	Sub-pN to tens of nN (depends on bead size and position)	≈0.1 pN to a few hundred pN
Probe geometry and contact area	Glass pipette, inner radius ≈1–5 µm; contact area set by the pipette radius and by the length of membrane aspirated	Sharp tips (radius ≈10–50 nm) to colloidal probes (≈1–10 µm); contact area set by tip radius and indentation depth	Ferromagnetic beads ≈4–5 µm bound to cell-surface receptors; contact area set by bead radius and degree of embedding	Beads ≈1–10 µm in a chamber; contact area set by bead radius	Beads ≈1–5 µm; contact area set by bead radius and by the degree of bead immersion in the cell surface
Spatial specificity and depth of deformation	Low: whole cell or µm-scale membrane patch; global deformation of membrane, cortex and cytoplasm	High: nm–µm lateral resolution; indentation depth from tens of nm to µm (cortex to cytoplasm)	Intermediate: bead–cell contact area; response usually averaged over many beads, although mapping is possible	Intermediate: single bead–cell contacts, many measured in parallel	High: user-defined site with sub-µm positioning; shallow deformation (membrane and cortex)
Temporal window/frequency bandwidth	Quasi-static to seconds; creep and relaxation protocols	Quasi-static to ≈1 kHz; higher with small dedicated cantilevers	Broad frequency window, ≈10⁻^2^ to 10^3^ Hz	Seconds to minutes; oscillatory protocols possible	Up to ≈1 kHz; extended over about five decades with time-shared traps
Throughput	Low (single cells)	Low to intermediate (single cells; force mapping; automation)	High (hundreds to thousands of beads in parallel across different cells)	High (many beads in parallel across different cells)	Low (single cells)
Simultaneous imaging of the probed site	Brightfield standard; compatible with fluorescence on standard microscopes	Routine on inverted microscopes; integrated fluorescence/confocal configurations available but require dedicated setups	Requires dedicated MTC–fluorescence/confocal setups	Optical bead tracking inherent; fluorescence imaging of the cell constrained by chip geometry	Readily combined with fluorescence/confocal on the same microscope; correlative SEM possible
Calibration requirements	Pressure transducer calibration; accurate measurement of the pipette inner radius	Cantilever spring constant and deflection sensitivity	Magnetic field and torque calibration; bead magnetization	Force calibration per bead and per position within the chamber	Trap stiffness calibrated in the cellular environment; sensitivity to refractive index and axial bead position
Probe–cell coupling	Suction seal on the membrane; no receptor specificity	Direct indentation, or functionalized probes for specific receptors	Receptor-specific attachment; variable degree of bead embedding	Bead–receptor attachment	Non-specific or receptor-specific bead attachment; immersion angle sets the contact geometry
Main artifacts and sources of variability	Strongly geometry- and model-dependent analysis; best suited to non-adherent cells, so adherent cells must be detached	Choice of contact-mechanics model; tip geometry; substrate effects in thin cell regions; loading-rate and indentation-depth dependence	Heterogeneous bead binding and embedding geometry affect the apparent stiffness; population averaging can mask cell-to-cell variability	Predominantly axial forces; position-dependent force amplitude; possible acoustic heating	Photothermal heating and photodamage (wavelength- and power-dependent); limited maximum force; low throughput
Selected references	Evans (1973); González-Bermúdez et al. (2019) ; Hochmuth (2000); Wu et al. (2018)	Delgado et al. (2024); Gisbert et al. (2024); Hecht et al. (2015); Krieg et al. (2019)	Fabry et al. (2001); Park et al. (2010); Wang et al. (1993); Zhang et al. (2017)	Bergamaschi et al. (2024); Nguyen et al. (2021); Sitters et al. (2015); Sorkin et al. (2018)	Ayala et al. (2016); Pontes et al. (2011); Pompeu et al. (2021); Icard-Arcizet et al. (2008); Ashraf et al. (2026)

*Operational criteria.* **Spatial specificity** — high: force applied at a user-selected subcellular site of ≤1–2 µm; intermediate: µm-scale probe–cell contacts that are not individually targeted; low: whole cell or large membrane patch. **Throughput** — low: tens of cells per experiment; intermediate: tens to hundreds; high: hundreds or more measured in parallel. **Depth of deformation** refers to the structural levels engaged by the probe, from the membrane and cortex alone to the underlying cytoplasm. Values are typical orders of magnitude taken from the cited references and depend strongly on the specific implementation; they are intended for comparison between techniques rather than as absolute specifications

Micropipette aspiration (MA) was among the first techniques used in the 1970 s by Evans and colleagues to measure the elastic properties of RBCs (Evans 1973). By applying a controlled suction pressure through a glass pipette and measuring the length of cell surface drawn in, one can estimate cell elastic parameters. Suction pressures typically range from a few pascals (Pa) to several kPa, which, for pipette radii of a few micrometers, correspond to forces from the piconewton to the nanonewton range (Hochmuth 2000; Gonzalez-Bermudez et al. 2019). It remains a robust method for non-adherent cells with simple geometries. However, its limitation is spatial, as it probes large membrane and cytoplasm regions under global deformation and cannot properly resolve the local, subcellular responses at the surface of adherent cells (Fig. 1; Table 1).

Atomic force microscopy (AFM) brought nanometer spatial resolution to cell mechanics, enabling the mapping of mechanical properties across cell surfaces with subcellular precision (Krieg et al. 2019). AFM heads are routinely mounted on inverted optical microscopes, and integrated AFM–fluorescence configurations are well established and continue to be developed (Krieg et al. 2019; Delgado et al. 2024). Its practical limitation in this context is therefore not an inability to image, but the fact that high-resolution fluorescence imaging of the exact deformation site, beneath the cantilever and during the measurement, requires dedicated instrumentation and is less routinely implemented than AFM alone. In addition, the measured response depends on tip geometry, indentation depth, and the contact-mechanics model used for analysis (Hecht et al. 2015; Gisbert et al. 2024) (Fig. 1; Table 1).

Magnetic twisting cytometry (MTC) probes many cells simultaneously, something that single-probe techniques cannot do (Fabry et al. 2001; Wu et al. 2018). In this approach, a magnetic field twists ferromagnetic beads of a few micrometers bound to cell-surface receptors. The applied load is usually expressed as a torque per bead volume (specific torque, in Pa) rather than as a force, and it can be oscillated over a broad frequency window, from approximately 10⁻^2^ to 10^3^ Hz (Wang et al. 1993; Fabry et al. 2001). This capacity to probe many cells at once is what made MTC central to population-level rheological studies, and much of what we know about the power-law viscoelastic behavior of cell cortices comes from these experiments. Spatial mapping of cytoskeletal stiffness and loss tangent within individual cells by MTC reveals that mechanical properties are not uniform across the cell surface, as regions of high F-actin density and high cytoskeletal prestress are stiffer and more solid-like (Park et al. 2010). The tradeoff, however, is a loss of spatial specificity and the complexity of combining these approaches with live fluorescence imaging, which requires dedicated instrument integration (Zhang et al. 2017) (Fig. 1; Table 1).

Acoustic force spectroscopy (AFS) addresses part of this tradeoff by combining the multiplexing capability of MTC with single-particle precision and forces ranging from the sub-piconewton to tens of nanonewtons (Sitters et al. 2015; Bergamaschi et al. 2024). In an AFS setup, a piezoelectric transducer generates a bulk acoustic standing wave in a microfluidic chamber, applying oscillatory forces simultaneously to many surface-attached beads, enabling frequency-dependent measurements across entire cell populations in parallel, with a throughput inaccessible to single-bead techniques (Sitters et al. 2015; Sorkin et al. 2018). Bead positions are tracked optically, but high-resolution fluorescence imaging of the cell at the deformation site is constrained by the chip geometry and is not yet routinely implemented (Fig. 1; Table 1).

OT occupy a distinct position in this landscape. Developed by Arthur Ashkin, whose work was recognized with the Nobel Prize in Physics in 2018, OT trap a micron-scale dielectric bead in a tightly focused laser beam and use that bead as a force transducer, allowing piconewton-precision force application at a defined location on the cell surface (Ashkin et al. 1986; Catala-Castro et al. 2022). The bead can be pressed against the cell surface and withdrawn at controlled velocity to extract a membrane tether, or oscillated laterally to probe the viscoelastic response of the membrane-cortex complex at a chosen frequency. Because OT systems are built around inverted microscopes, force measurement and live imaging, for example of fluorescently labeled cellular components, can be performed simultaneously in the same experiment (Icard-Arcizet et al. 2008; Leijnse et al. 2022; Ashraf et al. 2026). This is comparatively straightforward to implement with OT, but not exclusive, as integrated AFM- and MTC-fluorescence configurations are also available (Zhang et al. 2017; Delgado et al. 2024). Combining OT with brightfield and scanning electron microscopy (SEM) on the same sample extends this correlative capacity even further (Pompeu et al. 2021). More recently, force-feedback systems have extended OT-based measurements to the direct quantification of cell deformation, stiffness, and creep response under precisely controlled constant forces in the piconewton range, applicable to both adherent and suspended cells from a single measurement (Rodenburg et al. 2025; Rodenburg and Eeftens 2025). These advantages come at a cost: OT measurements are low-throughput, the accessible forces (up to a few hundred piconewtons) limit the magnitude of deformations that can be imposed, the trap must be calibrated in the cellular environment, and laser exposure can cause photothermal heating and photodamage (Neuman et al. 1999; Peterman et al. 2003) (Fig. 1; Table 1).

Beyond force range, spatial resolution and throughput, several features determine what each technique actually measures and should be considered when comparing results. These include the accessible temporal bandwidth or frequency window; the probe geometry and contact area, which set the volume of material that is deformed and the depth of deformation, from the membrane and cortex to the cytoplasm; the calibration requirements (cantilever spring constant, trap stiffness in the cellular environment, and acoustic or magnetic force calibration); the coupling between probe and cell, which for bead-based methods depends on the receptor used and on the degree of bead immersion (Kamgoue et al. 2007; Pontes et al. 2011; Ayala et al. 2016); and technique-specific artifacts, such as photothermal heating and photodamage in OT or substrate effects in AFM indentation of thin cell regions. Because these factors differ between techniques, differences in reported values often reflect the measurement geometry and the structural level probed as much as the biology (Wu et al. 2018; Krieg et al. 2019). Figure 1 and Table 1 summarize these characteristics. No single technique is therefore sufficient. A complete mechanical and structural characterization of the cell surface would in principle require several of these approaches in combination, and in practice, the question asked determines the choice made.

## Force as a readout (seconds): biophysical parameters of the cell surface

The techniques previously introduced answer specific questions: (i) How much does the cell surface resist stretching? (ii) How much does it resist bending? (iii) How tightly is the plasma membrane attached to the underlying cortex? (iv) How solid- or fluid-like is the whole interface at a given timescale? Thus, poking the cell surface gives answers in the form of measurable parameters. Static parameters extracted at or near mechanical equilibrium (membrane tension, bending rigidity, and MCA) are presented in the “Static mechanical parameters: membrane tension, bending rigidity, and membrane-cortex adhesion” section. The frequency-dependent viscoelastic character of the cortex, described within the framework of soft glassy materials, is discussed in the “Cortical viscoelasticity: the cell surface as a soft glassy material” section. Finally, the “Red blood cells: paradigmatic model for membrane mechanics and disease” section presents the RBC as a model in which these parameters connect directly to disease.

### Static mechanical parameters: membrane tension, bending rigidity, and membrane-cortex adhesion

**Fig. 2:**
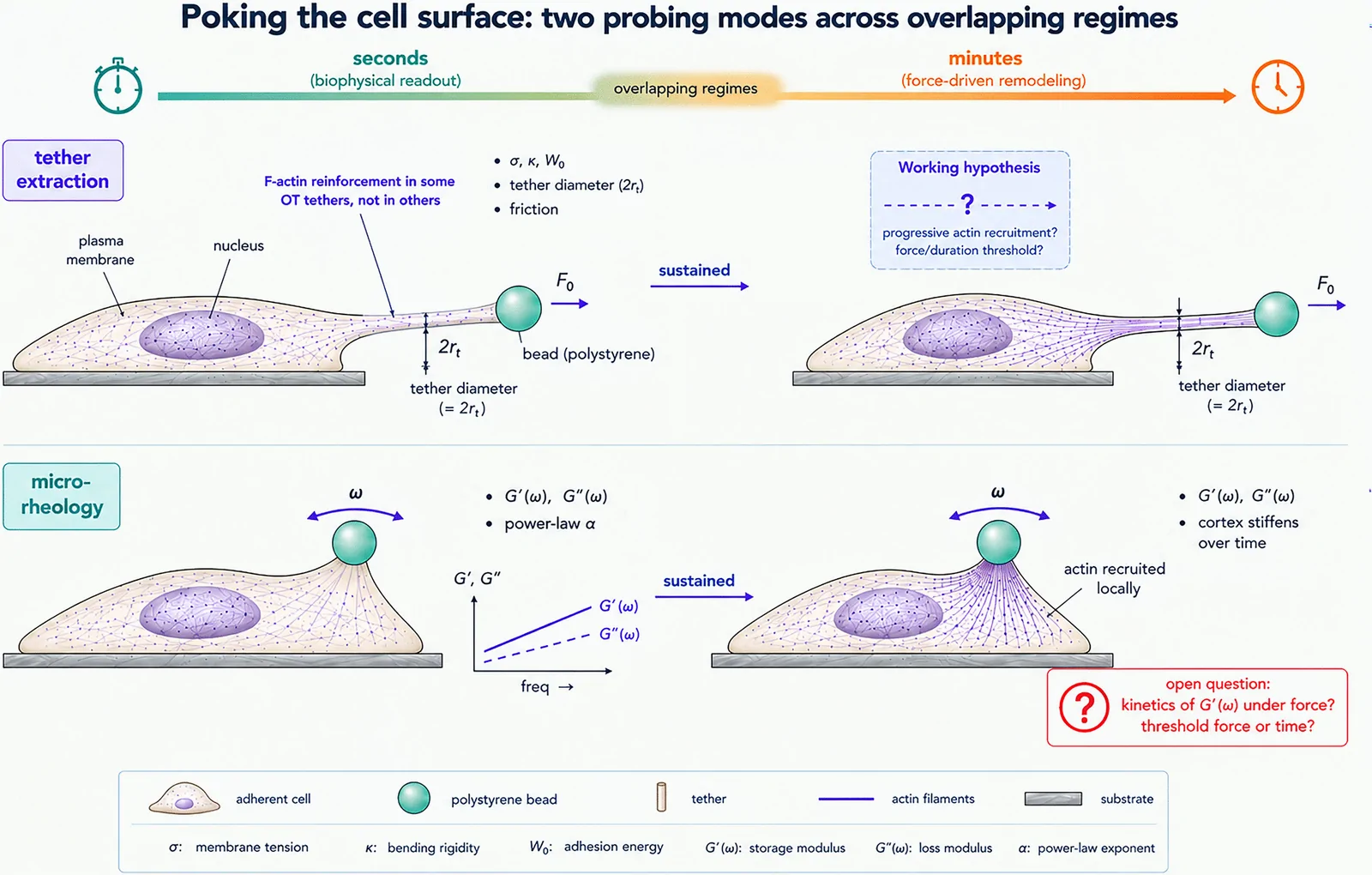
Poking the cell surface: two probing modes across overlapping regimes. The horizontal timeline is drawn as a gradient with a central overlap zone to indicate that the biophysical readout regime (seconds) and the force-driven remodeling regime (minutes) overlap substantially; they denote dominant experimental protocols rather than a sharp temporal boundary. Tether extraction (top row): a polystyrene bead held in the trap is used to pull a membrane tether at force $F_0$, yielding membrane tension (σ), bending rigidity (κ), membrane-cortex adhesion energy ($W_0$), tether diameter ($2{r}_{t}$) and membrane-cortex friction. F-actin has been detected in OT-extracted tethers from several adherent cell types under specific experimental conditions, but not in others (“F-actin in tethers: evidence and controversy” section). When force is sustained, whether actin is progressively recruited into the tether, and whether a force or duration threshold exists, have not been tested; this is indicated by the dashed arrow, the question mark and the “Working hypothesis” box. Microrheology (bottom row): an OT-trapped bead is oscillated at frequency ω, yielding the storage modulus $G^{\prime}(\omega)$, the loss modulus $G^{\prime\prime}(\omega)$ and the power-law exponent α. Sustained local stress stiffens the cortex, with local actin recruitment at the site of oscillation over minutes; the kinetics of this stiffening and the existence of a force or time threshold remain open questions. Symbols and abbreviations are defined in the key at the bottom

When an OT-trapped bead attached to the plasma membrane of an adherent cell is pulled away at a constant velocity, a thin cylindrical membrane nanotube, or tether, is extracted from the surface (Fig. 2). Tether formation occurs because the membrane is under tension and tends to minimize its area, but cannot collapse to zero tether radius because of its resistance to bending: the balance between membrane tension σ and bending modulus κ determines the equilibrium tether radius ${r}_{t}$ and the force ${F}_{0}$ required to sustain the nanotube. The relationships between these quantities, derived from the free energy of a cylindrical membrane, are ${F}_{0}=2\pi \sqrt{\left(2\sigma \kappa \right)}$ and ${r}_{t} = \sqrt{(\kappa/2\sigma)}$ (Derenyi et al. 2002; Nussenzveig 2018).

Measuring both ${F}_{0}$ and ${r}_{t}$, the latter by SEM of pre-formed tethers, allows the simultaneous and independent determination of σ and κ (Pontes et al. 2011, 2013; Pompeu et al. 2021) (Fig. 2). This is critical, as most studies in the literature report only ${F}_{0}$ and assume a standard value of κ to estimate membrane tension, an assumption that is problematic because κ varies between cell types, membrane compositions, and physiological states (Hochmuth et al. 1996; Pontes et al. 2011, 2013; Hissa et al. 2013, 2017; Ayala et al. 2017). ${F}_{0}$ alone is an indirect and entangled readout, as it can reflect changes in σ, in κ, or in both simultaneously. Disentangling these contributions requires the independent measurement of ${r}_{t}$, which is what makes the combined OT and SEM approach methodologically distinct (Pompeu et al. 2021). Because ${r}_{t}$ (~ 50–150 nm) lies below the resolution limit of conventional optical microscopy, the correlative approach has been used to obtain both parameters, although label-free quantitative phase imaging combined with OT, as well as super-resolution methods, are being explored as alternatives that would avoid fixation (Soares et al. 2022).

The effective membrane tension measured by tether extraction is not only the in-plane tension of the lipid bilayer. It is the apparent tension that integrates two distinct physical contributions. The first is, indeed, the in-plane tension ${\gamma }_{m}$, which reflects the actual stretching state of the lipid bilayer and is modulated by osmotic pressure, membrane reservoir availability (caveolae, ruffles, and membrane folds), and forces generated by actin polymerization or actomyosin contractility against the membrane (Roffay et al. 2021; De Belly et al. 2023) (Fig. 2). Membrane reservoirs function as physical buffers of ${\gamma }_{m}$. They flatten and release stored area when membrane tension rises, and reassemble when membrane tension decreases, providing a first line of mechanical homeostasis that is passive, local, and ATP-independent (Raucher and Sheetz 1999a; Sinha et al. 2011). Caveolae, in particular, respond within seconds to acute mechanical stress by rapid disassembly (Sinha et al. 2011). In addition, osmolarity changes couple directly to ${\gamma }_{m}$ through passive water flows that alter the bilayer stretching state, creating mechanical feedback in which a volume increase raises ${\gamma }_{m}$ and activates volume regulatory responses that restore tension toward its setpoint (Roffay et al. 2021). The second physical contribution to the effective membrane tension is the MCA energy, ${W}_{0}$, the energy cost per unit area of detaching the bilayer from the underlying actin cortex (Dai and Sheetz 1999; Sheetz 2001). The total apparent tension is therefore $\sigma = {\gamma }_{m} + {W}_{0}$. The dominance of ${W}_{0}$ in adherent cells has been demonstrated directly. Tether forces in intact cells are 2–4 times higher than in plasma membrane vesicles (Pontes et al. 2013) or blebs (Dai and Sheetz 1999) from the same cell.

***Box 1**** Membrane tension versus cortical tension*. It is important to distinguish effective membrane tension, measured by tether extraction, from cortical tension, which emerges from actomyosin contractility and governs cell rounding pressure and global cell shape (Salbreux et al. 2012). They are related but distinct parameters: cortical tension reflects the active mechanical state of the actin cortex, whereas effective membrane tension reflects the composite state of the bilayer-cortex interface as defined above. Tether extraction measures membrane tension specifically, and not cortical tension.

MCA is not uniformly distributed across the cell surface. Instead, it is concentrated in domains sensitive to cholesterol content, lipid phase, and signaling state, meaning that ${W}_{0}$ varies spatially (Chadda et al. 2007; Chugh et al. 2017). MCA is maintained by a specialized set of proteins, particularly the ezrin-radixin-moesin (ERM) family, the ankyrin-spectrin system, and class I myosins, all of which physically bridge membrane domains to cortical actin filaments (Diz-Munoz et al. 2010). Their assembly state is tightly regulated by signaling, as phosphorylation-dependent activation of ezrin increases tension (Liu et al. 2012), while its inactivation or knockdown reduces it (Diz-Munoz et al. 2010). Class I myosins also modulate membrane tension, as overexpression of Myo1a or Myo1e increases it (Nambiar et al. 2009). Myo1e responds to increases in membrane tension as a mechanosensor. As tension rises and clathrin-mediated endocytosis slows globally, Myo1e is recruited to endocytic sites to build a more effective branched actin network (Smith et al. 2026).

Disruption of F-actin, inhibition of Arp2/3, and Cdc42 knockdown all reduce membrane tension (Pontes et al. 2011; Bretou et al. 2014; Diz-Munoz et al. 2016), while the effect of myosin II inhibition by blebbistatin is context-dependent, decreasing tension in resting cells (Bretou et al. 2014; Ayala et al. 2017) but increasing it in actively migrating or spreading cells where actin polymerization forces dominate (Gauthier et al. 2011; Houk et al. 2012; Lieber et al. 2013). The lipid composition of the bilayer also modulates membrane tension. Reducing plasma membrane cholesterol with methyl-beta-cyclodextrin increases tension in fibroblasts (Hissa et al. 2013), embryonic kidney cells (Khatibzadeh et al. 2012), and cardiomyocytes (Hissa et al. 2017), with the increase reflecting both direct changes in bilayer mechanical properties and concomitant cytoskeletal rearrangements. These findings highlight that membrane tension belongs to the bilayer-cortex system as a whole and cannot be reduced to a cytoskeletal readout.

Bending rigidity κ has received far less attention than membrane tension, largely because it has long been treated as a fixed material property of the bilayer, typically assumed to lie around 20–25 kBT, and used as a constant in calculations, without experimental verification. This assumption does not hold, as direct measurements in living adherent cells, by tether extraction with simultaneous determination of tether radius, reveal that κ spans from a few tens to a few hundreds of kBT, varying substantially across cell types and physiological states (Pontes et al. 2011, 2013; Hissa et al. 2013, 2017; Ayala et al. 2017; Soares et al. 2020). For example, resting microglia and macrophages have markedly higher bending rigidity than neurons, but this difference reverses when microglia and macrophages are activated by lipopolysaccharides (LPS) (Pontes et al. 2013). Values measured from intact cells differ from those of plasma membrane vesicles derived from the same cells (Pontes et al. 2013; Steinkuhler et al. 2019). This distinction directly implicates the cytoskeleton in setting an effective bending rigidity of the cell surface. In intact cells, κ reflects both the intrinsic mechanics of the lipid bilayer and the additional constraint imposed by cytoskeletal attachment. When that attachment is disrupted by cytochalasin D, κ increases, moving toward values characteristic of pure lipid membranes (Pontes et al. 2013; Ayala et al. 2017). Lipid composition adds a further layer of regulation: in fibroblasts, cholesterol depletion increases both κ and σ, with changes in bending rigidity correlating with shifts in membrane order (Hissa et al. 2013; Steinkuhler et al. 2019). These findings reframe κ as a dynamic, regulated parameter that encodes the mechanical state of the cytoskeleton-membrane interface, and not a constant of the bilayer. How κ is regulated, and whether it changes during cellular processes or in disease, remains an open and important question in the field.

The maximum force applied to the cell surface before tether initiation, ${F}_{m}$, carries information beyond what the equilibrium force ${F}_{0}$ alone captures. While ${F}_{0}$ reports on the balance between membrane tension and bending rigidity, ${F}_{m}$ reflects the additional cost of locally rupturing the MCA to initiate tether formation (the energy barrier that must be overcome before the bilayer can detach from the underlying cortex). It is therefore particularly sensitive to the local density and affinity of MCA proteins at the extraction site, and systematically exceeds ${F}_{0}$ in proportion to the strength of that local adhesion (Pontes et al. 2017a). Most studies in the literature report ${F}_{0}$ but not ${F}_{m}$, leaving this parameter largely unexplored as a local readout of MCA organization.

The velocity dependence of tether force reveals a second role for MCA proteins, one that goes beyond static equilibrium. While $W_0$ reflects the adhesion energy that MCA proteins store per unit area, their density also determines the frictional resistance to lipid flow. As lipids flow toward the growing tether, MCA proteins act as fixed obstacles to this flow, generating a viscous drag proportional to extraction velocity (Hochmuth et al. 1996; Brochard-Wyart et al. 2006). Pulling tethers at multiple velocities makes it possible to quantify this membrane-cortex friction coefficient. It reports on the density and turnover dynamics of MCA linkers in a way that static force measurements cannot. The same friction that resists lipid flow during tether extraction also determines how rapidly tension perturbations propagate across the cell surface. A parameter directly measurable by OT, in other words, connects to a broader question, developed in the following paragraphs, about how mechanical information travels within the cell.

Whether membrane tension acts as a global or local regulator of cell behavior has been the subject of active debate. Several studies support rapid global propagation. The first evidence came from neutrophils, where increasing membrane tension at one pole inhibited leading-edge formation and signaling at the opposite pole within seconds (Houk et al. 2012). However, Shi et al. (2018) challenged this view by demonstrating that in slow-migrating cells, changes in membrane tension between two tethers 5–15 µm apart do not propagate over timescales of 10 min, while propagation occurs freely in blebs. The mechanism proposed (Cohen and Shi 2020) is that MCA proteins act as barriers to lipid flow, and that their density and distribution determine whether tension changes remain local or spread globally. Consistent with this framework, fast-migrating cells such as neutrophils and keratocytes show propagation on the timescale of seconds (Houk et al. 2012; Lieber et al. 2013), while slow-migrating cells do not (Shi et al. 2018). More recently, experiments combining optogenetics and dual-trap OT have clarified the conditions under which tension propagates. By activating actin protrusions locally while simultaneously measuring membrane tension at a distant site with OT, these experiments showed that mechanical stimuli acting on both the actin cortex and the plasma membrane generate rapid global tension propagation within seconds, while forces applied to the membrane alone do not, indicating that the membrane-cortex system, rather than the membrane alone, acts as a conduit for mechanical information transmission across the cell (De Belly et al. 2023).

How tension propagates determines the spatial reach of these signals. A rise in membrane tension that inhibits membrane curvature in one context drives vesicle fusion in another; the same tension that suppresses protrusion at the cell rear licenses it at the leading edge. What unifies these responses is that the cell surface is under continuous mechanical surveillance, and changes in membrane tension are converted into biochemical decisions with spatial and temporal precision. The most direct and immediate link is the activation of the mechanosensitive ion channel PIEZO1, which is gated by membrane tension and whose mechanical sensitivity is tuned by the resting tension of the cell membrane (Lewis and Grandl 2015; Luchtefeld et al. 2024). Membrane tension, through this direct coupling, drives mechanotransduction upstream. On longer timescales, membrane tension regulates a broad set of cellular processes, including endocytosis, exocytosis, phagocytosis, cell migration, polarity, and differentiation, through mechanisms that are increasingly well understood (Boulant et al. 2011; Gauthier et al. 2011; Houk et al. 2012; Masters et al. 2013; Pontes et al. 2017b; Bergert et al. 2021; De Belly et al. 2021).

In endocytosis, membrane tension acts as a physical barrier to the inward bending of the plasma membrane required for vesicle formation. Increased tension inhibits clathrin-mediated endocytosis, an antagonism that becomes acute during mitosis, when membrane tension rises and endocytosis is correspondingly suppressed (Raucher and Sheetz 1999b). At the molecular level, actin is recruited to counteract membrane tension and allow completion of clathrin-coated pit formation. When tension is elevated, coupling of actin filaments to clathrin provides the additional force needed to finish membrane bending (Boulant et al. 2011). Myo1e acts as a mechanosensor in this process (Smith et al. 2026). The transition of the clathrin coat from a flat to a curved geometry is regulated by the ratio of clathrin adaptors and by membrane tension, with higher tension stabilizing flat coats and lower tension favoring the curved configuration (Bucher et al. 2018). In vitro reconstitution has confirmed that low tension permits coat formation with extensive budding, moderate tension yields shallow pits, and high tension completely inhibits clathrin binding (Saleem et al. 2015). Beyond clathrin-mediated endocytosis, the relationship between tension and endocytosis forms a feedback loop. Among the multiple endocytic pathways operating simultaneously at the cell surface, the clathrin- and dynamin-independent CLIC/GEEC (CG) pathway is specifically and rapidly upregulated upon reduction of membrane tension, and its modulation directly sets steady-state membrane tension. Inhibiting the pathway decreases tension, while activating it increases tension (Thottacherry et al. 2018). This mechanochemical feedback is transduced by vinculin at focal adhesions, which senses tension changes and controls GBF1 recruitment to the plasma membrane to regulate CG endocytic rate. The passive mechanical response precedes this active one: upon sudden changes in cell shape or area, the membrane first forms passive invaginations that transiently store excess membrane area by minimizing elastic and adhesive energy, before active endocytic resorption restores membrane homeostasis over minutes (Kosmalska et al. 2015).

In exocytosis, the relationship with membrane tension runs in the opposite direction. During cell spreading, depletion of membrane reservoirs triggers a sudden tension increase that acts as a mechanical cue driving exocytosis of GPI-anchored protein vesicles to expand plasma membrane area (Gauthier et al. 2011). More broadly, membrane tension determines the mode of vesicle fusion. Cells with higher tension preferentially undergo complete vesicle fusion (full-collapse), while cells with lower tension favor partial fusion (kiss-and-run), a principle first proposed by modeling (Chizmadzhev et al. 2000; Shillcock and Lipowsky 2005) and confirmed experimentally in chromaffin cells (Bretou et al. 2014) and neurons (Stevens and Williams 2000). In cells with high membrane-cortex friction, such as adrenal chromaffin cells, tension perturbations remain spatially confined, constraining vesicle recycling to local kiss-and-run fusion, while in cells with low friction, such as the giant synaptic terminals of goldfish retinal bipolar neurons, tension perturbations propagate rapidly, supporting spatially segregated exo- and endocytosis through full fusion (Gomis Perez et al. 2022).

In phagocytosis, membrane tension orchestrates the sequential phases of pseudopod extension. The initial rapid phase uses actin polymerization to push the membrane forward while membrane reservoirs buffer tension increases; once reservoirs are depleted, tension rises sharply and functions as a signal that reorganizes Rac1, phosphoinositide distributions, and actomyosin contractility, while simultaneously triggering exocytosis of GPI-anchored protein vesicles to provide additional membrane area for continued particle internalization (Masters et al. 2013).

In cell migration and polarity, membrane tension functions as a long-range inhibitory signal that coordinates protrusion activity across the cell surface. Increasing tension at the leading edge, through actin polymerization pushing against the membrane, inhibits the formation of new protrusions elsewhere in the cell by suppressing Rac1 and SCAR/WAVE signaling, thereby confining protrusive activity to the leading edge and maintaining directional polarity (Houk et al. 2012). At the level of adhesion organization, membrane tension controls the positioning of new adhesion sites at the leading edge. Tension oscillations that periodically compress and relax the lamellipodium determine the spatial pattern of adhesion row formation during 2D migration (Pontes et al. 2017b). Migrating cells also display a consistent spatial asymmetry in MCA, high at the cell rear, where it couples actomyosin contraction to the membrane and suppresses protrusion, and low at the leading edge, where it allows deformation by actin polymerization and bleb-driven protrusion (Tsujita et al. 2021; De Belly and Weiner 2024). This same homeostasis functions as a mechanical tumor suppressor, as high membrane tension, maintained by ERM-mediated MCA, inhibits cancer cell migration and invasion by counteracting membrane curvature-sensing Bin/Amphiphysin/Rvs (BAR)-family proteins, and its disruption enables metastatic dissemination (Tsujita et al. 2021). The mechanochemical basis of long-range polarity coordination has recently been elucidated: Rac-based protrusions elevate membrane tension, which activates mTORC2 to stimulate Rho at the opposite pole of the cell, while Rho-mediated contractility induces cortical flow-based reorganization of membrane-cortex interactions that activates Rac distally, revealing a mutual long-range activation circuit that complements local inhibition to enable robust polarity (De Belly et al. 2023, 2026).

In differentiation, membrane tension regulates cell fate decisions at the earliest stages. Embryonic stem cells transitioning to a differentiated state detach the membrane from the underlying actin cortex, reducing effective membrane tension; conversely, maintaining high tension by enforcing MCA through constitutively active ezrin or synthetic inert MCA linkers locks cells in their pluripotent state (Bergert et al. 2021). Membrane tension also gates ERK-mediated signaling downstream of this mechanical state, linking the physical properties of the cell surface to the transcriptional programs that determine cell identity (De Belly et al. 2021).

In all these processes, tether pulling has been central to establishing the connections between membrane mechanics and cell biology, as it provides direct access to cell surface biophysical parameters that change during these events (Pontes et al. 2013; Pompeu et al. 2021). However, in recent years, the methodological landscape has expanded. The fluorescent tension probe Flipper-TR reports changes in lipid packing density via fluorescence lifetime imaging microscopy (FLIM), enabling non-invasive, spatiotemporally resolved measurements of membrane tension across multicellular environments, a resolution inaccessible to conventional tether pulling (Colom et al. 2018). Its key advantage is the ability to image tension dynamics in living tissues and multicellular systems without physical contact with the membrane. Rigorous calibration for each cell type is required for accurate absolute values, and the sensitivity to lipid composition must be carefully considered, as changes in cholesterol content or lipid phase can alter fluorescence lifetime independently of tension. Flipper-TR has already been applied to study endocytosis in yeast (Riggi et al. 2019), and rear retraction in migrating cells (Hetmanski et al. 2019), establishing it as a powerful complement to tether-based approaches. Combining force-controlled nanopipettes with Flipper-TR imaging, Luchtefeld et al. (2024) showed that localized mechanical stimulation induces a local pre-tension in the membrane that activates PIEZO1, and that this tension remains confined by cytoskeletal links.

Tether extraction gives access to three mechanically distinct but biologically coupled parameters: membrane tension *σ*, bending rigidity *κ*, and MCA friction. Each reports on a different layer of cell surface organization, and their combination constitutes a mechanical fingerprint whose biological significance extends across every process described in this section. Whether it changes in disease in ways that are measurable, quantitative, and sensitive is what gives it diagnostic potential, a possibility explored in the “Perspectives” section.

### Cortical viscoelasticity: the cell surface as a soft glassy material

Membrane tension and bending rigidity, extracted under quasi-static conditions, describe the apparent elastic response of the cell surface at the timescale of the measurement itself, without resolving how that response depends on the rate or frequency at which force is applied. Characterizing the dynamic, time-dependent mechanical behavior requires a different approach, one that subjects the cell surface to oscillatory deformations across a range of frequencies. When a material is deformed by an oscillatory force, its mechanical response has two components: one that stores energy elastically, like a spring, quantified by ${G}^\prime$, and one that dissipates energy through internal friction, like a viscous fluid, quantified by ${G}^{\prime\prime}$. Their ratio, the loss tangent, $\mathrm{t}\mathrm{a}\mathrm{n} \delta = G^{\prime\prime}/G^{\prime}$, indicates whether the material behaves more like a solid ($\mathrm{t}\mathrm{a}\mathrm{n} \delta < 1$) or more like a fluid ($\mathrm{t}\mathrm{a}\mathrm{n} \delta > 1$) at a given frequency. This is the domain of microrheology, and the response of living cells is neither purely elastic nor purely viscous, but something in between, and frequency-dependent in a characteristic way (Fabry et al. 2001) (Fig. 2).

Across a wide range of cell types and measurement techniques,  $G^{\prime}(\omega)$ and $G^{\prime\prime}(\omega)$ follow a power law in frequency, with exponent α typically between 0.1 and 0.3 (Fabry et al. 2001; Balland et al. 2006; Trepat et al. 2007; Ayala et al. 2016; Gomez et al. 2020a, b; Hang et al. 2021). Such behavior is a hallmark of soft glassy materials, structurally disordered systems near a glass transition. Within the soft glassy rheology framework, the exponent α is interpreted as a measure of the metastability of the cytoskeletal network: values near zero indicate solid-like elastic behavior whereas values approaching one indicate liquid-like viscous behavior. The generality of this behavior extends beyond the resting power-law exponent: when cells are subjected to a transient mechanical stretch, the cytoskeleton fluidizes (softening and increasing α) regardless of cell type or of the specific molecular target of pharmacological treatments applied, before slowly resolidifying over tens of seconds to minutes (Trepat et al. 2007). This response was consistent across the cell types and conditions tested, leading Trepat and collaborators (2007) to describe it as a universal physical response of living cells to stretch, and it supports viewing soft glassy behavior as an active and regulated cellular state rather than a fixed material property (Fig. 2).

The power-law dependence on frequency does not come from any single cellular component but from the composite architecture of the cell surface, the actomyosin cortex, the MCA linkers, and the underlying cytoplasm, all of which contribute to the response in ways that depend on the timescale of the applied perturbation (Stamenovic et al. 2007; Hang et al. 2021). At short timescales, the cortex response is dominated by viscous dissipation from individual filament fluctuations and lipid bilayer sliding, a friction mechanism analogous to the one that generates the velocity-dependent drag quantified by tether extraction (“Static mechanical parameters: membrane tension, bending rigidity, and membrane-cortex adhesion” section). At longer timescales, elastic contributions from the crosslinked actomyosin network dominate. The frequency at which $G^{\prime\prime}$overtakes $G^\prime$, also called the transition frequency, marks the crossover between these regimes and varies with cell type, cytoskeletal state and pathological condition; it has therefore been proposed as a candidate mechanical marker of cell phenotype (Rigato et al. 2017; Hang et al. 2022) (Fig. 2). Like the power-law exponent, however, its value depends on the accessible frequency window and on the measurement geometry, so comparisons are most informative within a given technique and protocol.

Measurement of these parameters at the cell surface has been performed with several tools, each probing a distinct physical window. AFM has been central to this effort. By applying oscillatory indentations across a range of frequencies, it can map the storage and loss moduli across the cell surface with sub-micrometer spatial resolution (Radmacher 1997; Rigato et al. 2017). Loading rate and indentation depth are critical considerations. At low loading rates, shallow indentations probe the cortex and give access to its stiffness and viscoelastic character, while higher loading rates engage the response of the cytoplasm, which is softer and more liquid-like (Gisbert et al. 2024). The power-law exponent measured at the cortex is lower than that of the cell interior, indicating a more elastic response (Rigato et al. 2017; Hang et al. 2021). Probe tip size matters as well, since smaller tips resolve the mechanics of individual stress fibers and focal adhesions, while larger tips report an average over multiple structural elements, with modulus values differing by orders of magnitude between these scales (Hecht et al. 2015). Applied systematically across several cell lines ranging from healthy fibroblasts to cancer cells, AFM measurements in the time domain reveal a ubiquitous double power-law relaxation, with fast and slow exponents separated by a crossover timescale that varies with cell type and correlates with migration and invasiveness (De Sousa et al. 2020). Whether this crossover timescale reports on a distinct structural transition within the cortex remains an open question whose answer would extend the diagnostic reach of the parameters described in this review.

MTC offers something that AFM tips and single-bead techniques cannot: the ability to probe many cells simultaneously. By applying oscillatory torques to receptor-bound magnetic beads on the cell surface, MTC measures the viscoelastic response averaged over many bead-cell contacts, enabling population-level rheological characterization with high statistical power (Fabry et al. 2001). A dual-component model has been used to partition the contributions of the cortex and the cytoplasmic interior in MTC measurements, revealing that these two layers can respond differently to the same pharmacological perturbation (Laurent et al. 2003). Much of what is known about how actomyosin contractility and cytoskeletal drug treatments alter cortical viscoelasticity at the population level has come from MTC experiments. The tradeoff, as already discussed in this review, is a loss of spatial specificity and the difficulty of combining this approach with live fluorescence imaging.

AFS offers a complementary platform that combines the multiplexing capability of MTC with single-particle precision across a wide force and frequency range. By applying oscillatory acoustic forces to surface-attached beads in a microfluidic chamber, AFS-based microrheology resolves  $G^\prime(\omega)$ and $G^{\prime\prime}(\omega)$ across many cells simultaneously, detecting pharmacological perturbations and cell-to-cell mechanical heterogeneity (Sitters et al. 2015; Nguyen et al. 2021; Bergamaschi et al. 2024). The power-law exponent reported by AFS for fibroblasts ($\alpha \sim 0.1$) lies at the lower end of the range reported with other techniques ($\alpha \sim 0.1-0.3$ Fabry et al. 2001; Ayala et al. 2016; Hang et al. 2021)), albeit near its lower limit; a difference that may reflect the distinct frequency windows, bead sizes and contact geometries of each method.

OT offer a complementary capability. By oscillating a cell-surface-attached bead at controlled frequencies, OT-based microrheology gives access to $G^\prime(\omega)$ and $G^{\prime}(\omega)$ at a user-defined location on the cell surface (Ayala et al. 2016; Vos et al. 2024). Because OT systems are generally built around inverted fluorescence microscopes, force measurement and live imaging of fluorescently labeled cellular components can occur simultaneously in the same experiment. That capacity is what makes OT microrheology well suited to connecting mechanical parameters with structural states of the cortex in real time, directly observing how cytoskeletal organization relates to local viscoelastic behavior at the same site and moment (Icard-Arcizet et al. 2008). Applying this to different cell types reveals systematic differences that correlate with function. Fibroblasts and neurons exhibit relatively solid-like behavior ($\alpha \sim 0.2$) with storage moduli in the range of 50–140 Pa at physiologically relevant frequencies. Astrocytes, which serve a cushioning function in the central nervous system, are softer and more liquid-like, with a higher α and lower absolute moduli (Ayala et al. 2016). Differences in cytoskeletal architecture, crosslinking density, and myosin activity are likely to underlie this contrast, which may be relevant to how cells perform their mechanical roles in their respective tissues. Beyond oscillatory protocols, extension-jump experiments that apply a step force and record the subsequent relaxation give access to viscoelastic parameters in the time domain, revealing relaxation processes across multiple timescales that frequency-domain measurements alone cannot fully resolve (Gironella-Torrent et al. 2024). Recent implementations have extended this range substantially: time-shared OT microrheology, in which a single laser is split into two traps for simultaneous force and displacement measurements, covers five decades of frequency and has been applied to quantify age-dependent viscoelastic changes in organelles, intracellular compartments, and living organisms, extending OT microrheology beyond adherent cells to in vivo contexts (Catala-Castro et al. 2022).

A critical issue is the role of cell geometry. OT microrheology relates forces to deformations, but converting this relationship into the intrinsic shear modulus of the membrane-cortex material requires accounting for the geometry of the cell-bead contact: the bead immersion angle and the cell thickness beneath the bead both influence the measured response, and neglecting these corrections can introduce systematic errors of up to an order of magnitude (Kamgoue et al. 2007; Ayala et al. 2016). When these corrections are applied carefully (e.g., using SEM for the bead immersion angle and optical methods for cell thickness Ayala et al. 2016; Barreto et al. 2022)), results obtained with different approaches become more comparable and place the viscoelastic moduli of the membrane-cortex complex of adherent cells in the range of tens to hundreds of Pa.

The pharmacological dissection of these parameters has clarified the relative contributions of different cytoskeletal components. Disrupting actin polymerization with cytochalasin D reduces the viscoelastic moduli in a concentration-dependent manner (Ayala et al. 2017). The cortex becomes softer and more fluid-like under actin disruption, as indicated by decreased transition frequency and increased power-law exponent (Rigato et al. 2017; Flaschner et al. 2021). Inhibiting myosin II with blebbistatin reduces viscoelastic moduli in adherent cells, consistent with a primary role for myosin as a network crosslinker and tension generator (Ayala et al. 2017; Cordes et al. 2020). The role of myosin II in cortex rheology is, however, context-dependent: it fluidizes poorly crosslinked actin networks while solidifying dense, highly crosslinked cortices in spread cells, reflecting the opposing effects of myosin-driven filament sliding and myosin-mediated network prestress (Humphrey et al. 2002; Fischer-Friedrich et al. 2016; Cordes et al. 2020). The extent to which myosin penetrates the cortical layer also determines cortical tension and mechanical state, with deeper penetration generating greater tension and more solid-like behavior (Truong Quang et al. 2021). Inhibiting the Arp2/3 complex with CK666 drives the cortex toward a more elastic, solid-like configuration by converting the branched mesh into a more parallel filament arrangement, while formin inhibition reduces cortical elasticity by depleting the unbranched filament population (Fritzsche et al. 2016; Rigato et al. 2017; Flaschner et al. 2021). These experiments indicate that cortical viscoelastic properties are primarily set by actomyosin organization and dynamics, with the balance between branched and linear actin architecture, myosin penetration depth, and crosslinker turnover rates all contributing to where the cortex sits on the liquid-to-solid spectrum.

An important point that emerges from comparing these techniques concerns what is actually being measured. MTC, AFM, OT, and AFS all report power-law viscoelastic behavior with broadly comparable exponents, and this phenomenological convergence is commonly interpreted, within the soft glassy rheology framework, as reflecting the metastability of the cytoskeletal network (Fabry et al. 2001; Hang et al. 2021). Similar phenomenology, however, does not by itself establish identical microscopic origins. The techniques perturb different structural levels (membrane and cortex versus deeper cytoplasm), at different strain amplitudes and within different frequency windows, and both the exponent and the absolute modulus depend on probe size, indentation depth, oscillation amplitude, loading rate and the geometric corrections applied (Hecht et al. 2015; Hang et al. 2021; Gisbert et al. 2024). Absolute moduli can accordingly differ by orders of magnitude between techniques. These differences are not a failure of the techniques; at least in part, they reflect the structural heterogeneity of the cell surface, since different probes sample different structural levels (Park et al. 2010; Hang et al. 2021; Gisbert et al. 2024). Convergence at the phenomenological level, which is well supported, should therefore be distinguished from equivalence at the mechanistic level, which has not been established. Comparisons of power-law exponents or transition frequencies across studies are meaningful only when the frequency window, strain regime and measurement geometry are taken into account. With these caveats, the viscoelastic parameters accessible by these approaches extend the mechanical fingerprint of the cell surface beyond the equilibrium parameters of the “Static mechanical parameters: membrane tension, bending rigidity, and membrane-cortex adhesion” section, adding a time-dependent dimension to what force can reveal about cell state and disease.

### Red blood cells: paradigmatic model for membrane mechanics and disease

**Fig. 3:**
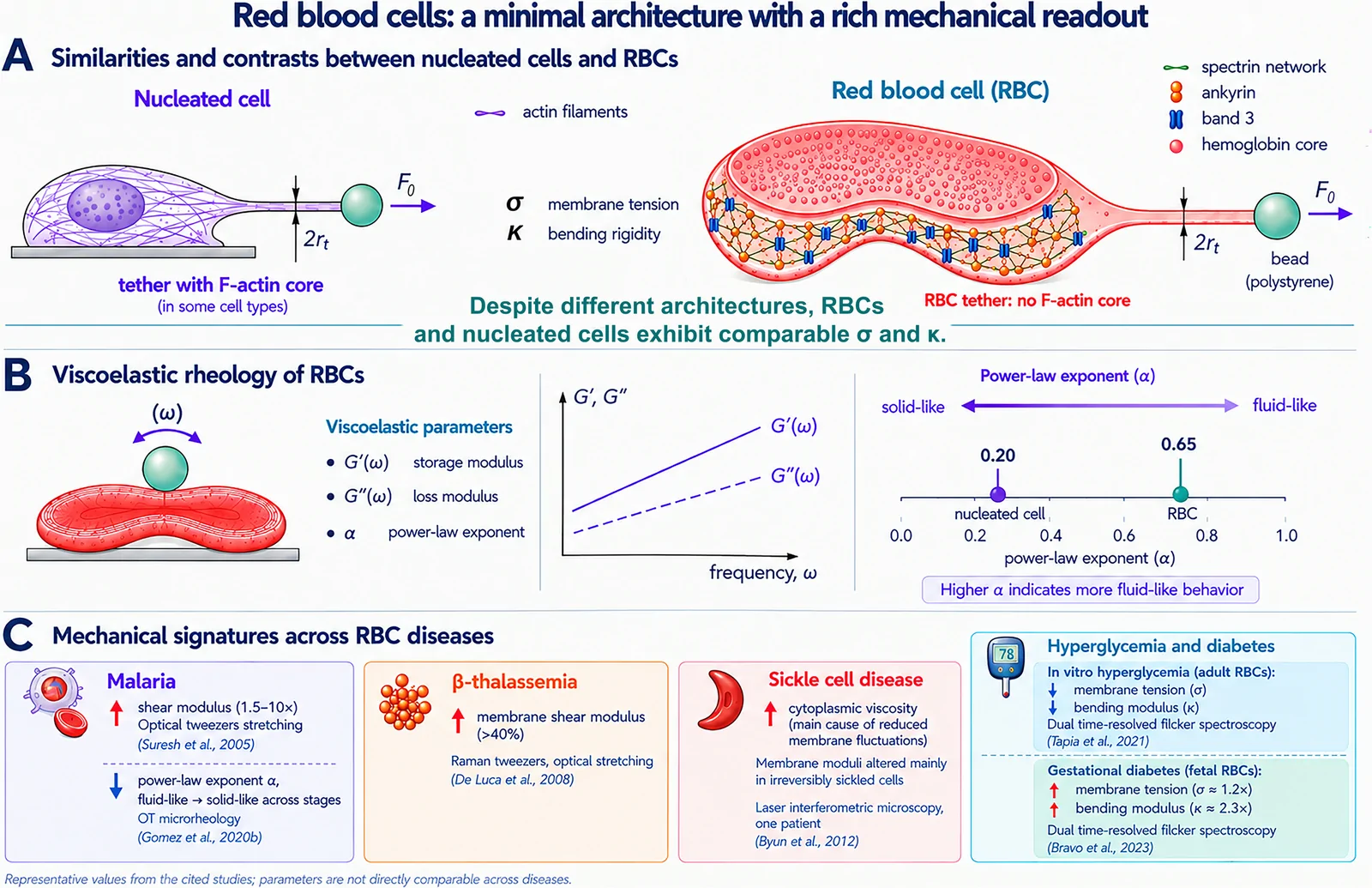
Red blood cells: a minimal architecture with a rich mechanical readout. **A** Tether extraction from a nucleated cell (left) and from an RBC (right), in both cases by pulling a bead with OT at force $F_0$, yielding a tube of diameter $2{r}_{t}$. The RBC membrane is supported by a spectrin network anchored to the bilayer through ankyrin and band 3, and encloses a concentrated hemoglobin solution. Unlike tethers from several nucleated cell types, in which an F-actin core has been detected under specific conditions, RBC tethers contain no detectable F-actin; despite these different architectures, σ and κ fall in a comparable range in the two cell types (Gomez et al. 2020a). **B** Microrheology of an RBC yields the frequency-dependent storage and loss moduli, $G^{\prime}(\omega)$ and $G^\prime(\omega)$, and the power-law exponent α. The RBC membrane-spectrin skeleton behaves as a markedly more fluid-like material ($\alpha \sim 0.65$) than the cortex of adherent nucleated cells ($\alpha \sim 0.20$). **C** Examples of mechanical alterations reported in malaria, β-thalassemia, sickle cell disease and hyperglycemia or diabetes. Each entry indicates the parameter measured, the direction of change, the principal technique and the reference. Values are representative of the cited studies; they were obtained with different techniques, and correspond to physically distinct measurements (shear modulus, cytoplasmic viscosity, membrane tension, bending rigidity, power-law exponent) that should not be compared directly with one another. Note that acute in vitro hyperglycemia (Tapia et al. 2021) and gestational diabetes (Bravo et al. 2023) shift σ and κ in opposite directions

RBCs hold a special place in the history of cell surface mechanics. They were the first cells on which membrane tether extraction was performed, in the pioneering experiments of Hochmuth and colleagues (Hochmuth et al. 1973, 1982; Hochmuth and Evans 1982), and these cells remain among the most characterized mechanically. The reason is structural. Mature RBCs lack a nucleus and most organelles; they consist of a lipid bilayer supported by a spectrin-actin network anchored to the bilayer through proteins such as ankyrin and band 3 (Mohandas and Gallagher 2008). Because their architecture is simpler than that of nucleated cells, RBCs are ideal for quantitative mechanical modeling, and their deformability has immediate physiological significance, as they must traverse capillaries smaller than their own diameter without rupturing (Fig. 3).

Unlike tethers from adherent cells, RBC tethers have not been found to contain filamentous actin. Confocal fluorescence microscopy of phalloidin-stained tethers detected no F-actin signal, consistent with the architecture of the RBC membrane-cytoskeleton itself (Gomez et al. 2020a). Yet, membrane tension and bending rigidity values measured from RBC tethers fall in the same range as those from nucleated cells, suggesting that these parameters reflect primarily bilayer properties modulated by membrane-cytoskeleton coupling, regardless of whether the underlying network is spectrin- or actin-based (Gomez et al. 2020a).

On the other hand, the rheological behavior of RBCs differs from that of adherent cells in a functionally meaningful way. Microrheology experiments reveal a power-law exponent $\alpha \sim 0.65$, markedly higher than the ∼0.2 typical of adherent cells, indicating a much more liquid-like response (Puig-De-Morales-Marinkovic et al. 2007; Gomez et al. 2020a, b; Barreto et al. 2022). This liquid-like character is a functional necessity, as it allows RBCs to deform reversibly and recover their shape after passing through the narrowest capillaries of the circulatory system. The contrast between fibroblasts ($\alpha \sim 0.2$, solid-like) and RBCs ($\alpha \sim 0.65$, fluid-like) suggests that the power-law exponent may reflect functional identity, with the degree of metastability of the underlying network tuned to the mechanical demands of each cell type (Fig. 3).

A more mechanistically resolved picture of RBC viscoelasticity emerges from force-relaxation experiments. After an extension jump applied with an optically trapped bead, the force relaxes following triple-exponential kinetics, with three well-separated timescales spanning four decades (0.01–100 s). The fast component (~ 0.02 s) corresponds to the relaxation of the membrane, whereas the intermediate (~ 4 s) and slow (~ 70 s) components likely arise from the dynamics of the membrane-spectrin skeleton and from cytosol viscosity, respectively (Gironella-Torrent et al. 2024). The three relaxation times are, moreover, correlated from cell to cell, suggesting that these contributions are coupled rather than independent.

The RBC surface also exhibits active, non-equilibrium fluctuations that no passive viscoelastic parameter captures, driven by ATP-dependent forces in the spectrin-actin cytoskeleton, a phenomenon known as flickering (Brochard and Lennon 1975; Yoon et al. 2009). These fluctuations are not purely thermal, as they violate the detailed balance expected at thermodynamic equilibrium, as demonstrated by the breakdown of displacement statistics in freely fluctuating RBCs (Turlier et al. 2016). The amplitude and energetics of flickering have been related to the metabolic state of the cell. Several studies report that ATP depletion dampens and slows the fluctuations, while cytoskeletal disruption increases their amplitude (Tuvia et al. 1998; Gov and Safran 2005; Turlier et al. 2016), although the extent of this ATP dependence has been debated (Evans et al. 2008). More recently, bead-free OT measurements, that track cell surface forces and displacements simultaneously, have enabled the construction of a mechano-dynamic phase space for RBCs under chemical perturbations, distinguishing between structural and metabolic states before morphological changes become apparent (Dorn et al. 2026). This active mechanical dimension is absent from tether-based and oscillatory microrheology approaches, which probe the passive viscoelastic response, and its integration with these parameters represents an open frontier for understanding how RBC mechanical dysfunction develops in disease.

The sensitivity of RBC mechanics to molecular-level alterations in the membrane-cytoskeleton has generated a substantial body of work on the biophysical signatures of hematological diseases, probed by a range of optical and force-based techniques. In *Plasmodium falciparum* malaria, the parasite exports proteins that remodel the spectrin-actin network and insert knob-like structures at the membrane surface, progressively stiffening the cell as the intraerythrocytic cycle advances (Cooke et al. 2001; Suresh et al. 2005). OT-based microrheology of infected RBCs shows stage-dependent changes that track this stiffening (Gomez et al. 2020b). Mechanistically, the stiffening corresponds to a progressive shift in viscoelastic character: as the parasite matures from the ring to the trophozoite and schizont stages, the power-law exponent decreases systematically, reflecting a transition from fluid-like to solid-like behavior that parallels the mechanical impairment of the cell (Gomez et al. 2020b). The storage and loss shear moduli, extracted from the same soft glassy rheology framework, capture this transition quantitatively. While the storage modulus rises progressively across maturation stages, the loss modulus falls, with RBCs infected by ring-stage parasites showing slightly more liquid-like behavior than healthy RBCs and those from trophozoite/schizont stages showing increasingly solid-like behavior, accompanied by increases in low-frequency surface viscosity and shape-recovery relaxation time (Gomez et al. 2020b). Notably, uninfected RBCs circulating in the same environment also stiffen, a phenomenon termed the bystander effect (Paul et al. 2013), which has been linked to increased intracellular cAMP levels rather than to direct parasite contact (Paul et al. 2019), and which is partially reversed by antimalarial treatment (Dorta et al. 2024).

The magnitude of this stiffening is considerable. The shear modulus rises by 1.5- to tenfold and Young’s modulus by up to tenfold as the parasite progresses through the intraerythrocytic cycle (Suresh et al. 2005), and direct force measurements of merozoite-erythrocyte attachment place the detachment force of post-viable merozoites at roughly 40 pN, reduced by more than 75% by heparin treatment (Crick et al. 2014). A gene-knockout study combined with OT further resolved which parasite proteins actually drive this attachment: the PfEBA/PfRH ligand family, not PfMSP1 as long assumed, dominates merozoite adhesion (Kals et al. 2024), providing a direct mechanical readout of this interaction. Perhaps the best illustration that tension itself, and not adhesion, is the protective variable comes from the Dantu blood group. Invasion rarely occurs above a threshold RBC tension, and this threshold applies to non-Dantu cells as well; because Dantu RBCs have a higher average tension, a greater proportion of them resist invasion. Notably, the strength of merozoite attachment measured by OT did not differ between Dantu and non-Dantu cells (Kariuki et al. 2020). The Dantu example recasts membrane tension from a passive elastic parameter into an active determinant of infection outcome.

While malaria illustrates how an external agent can remodel the RBC surface, the most direct correlation that molecular defects in the membrane-skeleton itself translate into measurable mechanical changes comes from hereditary membrane disorders. In hereditary spherocytosis, loss of the vertical linkages between the lipid bilayer and the spectrin network, involving ankyrin, band 3, protein 4.2 or spectrin, leads to membrane loss, a reduced surface-to-volume ratio and decreased deformability. In hereditary elliptocytosis, weakening of the lateral linkages between skeletal proteins, mainly the spectrin dimer–dimer association and the spectrin–actin–protein 4.1R junction, reduces membrane mechanical stability and causes membrane fragmentation; in both disorders, disease severity relates directly to the extent of surface area loss (An and Mohandas 2008). Micropipette measurements on individual cells from patients with spherocytosis and elliptocytosis showed that the reduction in membrane shear modulus scales with the fractional reduction in spectrin content and that the apparent bending stiffness is likewise reduced in proportion to spectrin density (Waugh and Agre 1988). The RBC therefore provided evidence for what “Static mechanical parameters: membrane tension, bending rigidity, and membrane-cortex adhesion” section develops for nucleated cells: that bending rigidity is a regulated property of the membrane-cytoskeleton interface rather than a constant of the bilayer. More recently, high-throughput morpho-rheological phenotyping of whole blood detected the reduced deformability of spherocytes directly, as a functional correlate of the underlying cytoskeletal mutation (Toepfner et al. 2018). Because the molecular lesion is known and its extent can be quantified, these disorders provide a natural reference system for connecting membrane-skeleton architecture to the mechanical parameters discussed in this review.

In sickle cell disease, analysis of dynamic surface fluctuations by laser interferometric microscopy in RBCs from a patient indicated that the markedly reduced fluctuations are mainly attributable to elevated cytoplasmic viscosity at ambient oxygen concentration, whereas the membrane mechanical moduli were altered predominantly in irreversibly sickled cells (Byun et al. 2012). In the same disease, Raman spectroscopy on sickle RBCs shows heightened susceptibility to deoxygenation specifically under mechanical stress, linking the biophysical and biochemical dimensions of the disease at the single-cell level (Liu et al. 2013). Optical folding and rotation measurements in a single polarized laser trap add further insight: RBCs from patients treated with hydroxyurea showed folding angles and rotation speeds closer to those of healthy cells than RBCs from untreated patients (Mohi et al. 2021). Beyond intrinsic cell surface alterations, components of the innate immune system that bind to sickle cells further modulate their elastic parameters, as demonstrated by the effect of mannose-binding lectin on sickle erythrocyte mechanics (Lima et al. 2025). More broadly, circulating metabolites can alter RBC surface mechanics. Incubation of healthy adult RBCs at increasing D-glucose concentrations decreased both membrane tension and bending modulus, as measured by dual time-resolved membrane fluctuation spectroscopy, an OT-based flicker technique (Tapia et al. 2021). Whether these mechanical changes produce distinct signatures in *σ*, *κ*, and *W*₀ measurable by tether extraction, and whether they alter viscoelastic parameters accessible by microrheology, remain unexplored. These are natural extensions of the approach described in this review.

β-Thalassemia offers a further test. In this disease, the primary defect lies not in the membrane-cytoskeleton scaffold itself but in the hemoglobin it encloses. Raman tweezers measurements on optically stretched β-thalassemic RBCs reported a membrane shear modulus more than 40% higher than that of healthy cells, together with a reduced oxygenation capability (De Luca et al. 2008), and laser tweezers Raman spectroscopy in a larger patient cohort confirmed characteristic hemoglobin-related spectral differences (Jia et al. 2018). That a hemoglobin-centered mutation propagates into a mechanical signature measured at the whole-cell level suggests that RBC deformability integrates cytoplasmic as well as membrane-skeletal contributions, consistent with the multi-timescale relaxation picture described above for healthy RBCs (Gironella-Torrent et al. 2024). Complementary measurements using cationic quantum dots alongside OT-based viscoelastic profiling further show a reduction in the negative surface charge of thalassemic membranes, pointing to altered band 3 and glycoprotein organization at the bilayer surface (Lima et al. 2020). Whether this electrical signature correlates with, or is independent of, the σ and κ parameters accessible by tether extraction has not been tested, and represents a natural point of convergence between electromechanical and tether-based characterization of the RBC surface.

Diabetes extends the metabolic dimension introduced above, beyond the acute glucose-tension coupling measured in vitro. A pilot study using dual-trap stretching showed that RBC deformability falls in type 2 diabetes and diabetic retinopathy, and that this reduction correlated inversely with the initial cell size, hinting at a geometric as well as a biochemical component to the mechanical impairment (Agrawal et al. 2016). The effect was not confined to adult disease: fetal RBCs from pregnancies complicated by gestational diabetes showed increased membrane tension and bending modulus, suggesting that mechanical alterations associated with glycemic dysregulation might already have been present before birth (Bravo et al. 2023). Acute in vitro hyperglycemia (Tapia et al. 2021) and gestational diabetes (Bravo et al. 2023) shift these parameters in opposite directions, indicating that the mechanical consequences of glycemic dysregulation depend on exposure duration, cell age and metabolic context, and cannot be predicted from glucose concentration alone. At the acute end of this spectrum, brief high-glucose exposure alone is sufficient to raise oxidative stress and reduce membrane elasticity in a dose-dependent manner (Singh et al. 2021), and laser tweezers Raman spectroscopy combined with multivariate classification separated diabetic from healthy erythrocytes with high accuracy on the basis of spectral (biochemical) rather than mechanical features, in a small single-center dataset, a result that awaits validation in independent cohorts (Lin et al. 2018). Together, these results suggest that glycemic state can modulate RBC surface mechanics, although the direction and magnitude of the effect vary with the biological context and the technique used, and that metabolic state may be reflected in the same parameters that this review treats as biophysical readouts of cell state.

Across several RBC diseases and conditions, cell surface mechanical parameters are altered in ways that can be quantified and, in some cases, interpreted mechanistically. These parameters, however, are obtained with different techniques, correspond to physically distinct quantities and are often derived from small numbers of donors, so they should not be compared directly across studies (Fig. 3). The same logic applies to nucleated cells in disease, the subject of the “Perspectives” section.

## Sustained force (minutes): remodeling of the cell surface

The previous section described the cell surface as a structure with measurable mechanical parameters, and force as a probe to obtain them. Tether pulling, however, can also be used to follow how the cell surface dynamically rearranges in response to an external force (Soares et al. 2022). When a localized force is sustained, the response of the cell surface increasingly includes active reorganization of the membrane-cortex interface. This is not a switch from a passive to an active regime at a defined time point. The “seconds” and “minutes” labels used in this review therefore denote dominant experimental regimes, brief readout versus sustained stimulation, which overlap substantially in time. The following subsections examine: (i) the evidence for F-actin within tethers (4.1); (ii) how sustained local force can reorganize the cortex and bias protrusive activity (4.2); and (iii) the hypothesis that force-extended membrane structures relate to TNTs and other intercellular connections (4.3). 

### F-actin in tethers: evidence and controversy

The classical description of membrane tethers treats them as membrane-dominated lipid tubes isolated from the actomyosin cortex (Raucher et al. 2000; Sheetz 2001). This description is useful for interpreting equilibrium force measurements, but it does not capture the full picture of tethers extracted from living adherent cells.

The presence of filamentous actin inside tethers has been a point of contention in the field. Several studies report F-actin enrichment within OT-extracted tethers from adherent cells (Pontes et al. 2011, 2013; Bornschlogl et al. 2013; Leijnse et al. 2022; Ashraf et al. 2026), while others do not detect it (Raucher et al. 2000; Gabella et al. 2014; Shi et al. 2018, 2022). These discrepancies probably reflect a combination of methodological and biological factors, including cell type, the tether-pulling protocol (pulling velocity, tether length and holding time), the mode of bead-membrane attachment, the cytoskeletal state of the cell and the sensitivity of the imaging method used to detect thin actin structures. Deviations of experimental force-extension curves from the predictions of the classical Helfrich-Canham description of a pure lipid tube provide additional, indirect support for cytoskeletal content in some tethers (Nussenzveig 2018). Taken together, current evidence indicates that F-actin has been detected in several OT-generated tethers under specific experimental conditions, but that it is not universally observed. Whether it is incorporated from the cortex, by which mechanism and under which conditions remain open questions (Fig. 2).

Tether-like structures containing F-actin arise physiologically across a range of cellular contexts, independent of any external manipulation. During cell migration, retraction fibers generated by actomyosin contractility at the trailing edge retain an actin core (Fan et al. 2022). During cell–cell dislodgement, a tether left behind on the surface of a stationary cell as a migrating neighbor pulls away is proposed to give rise to long-range intercellular bridges, a route to TNT formation discussed further in the “Toward intercellular connections: a working hypothesis linking tethers, protrusions, and tunneling nanotubes” section (Rustom et al. 2004; Onfelt et al. 2006; Ljubojevic et al. 2021; Zurzolo 2021; Dias et al. 2026). During wound healing, tethers formed at sites of partial cell-substrate detachment guide lamellipodial extension both in vitro and in vivo (Korkmazhan et al. 2022; Kennard et al. 2023). Despite their distinct origins, these structures are actin-containing tubes whose cytoskeletal organization determines their functional properties.

Supporting this view, the dynamic force behavior of OT-pulled tethers carries information about active cytoskeletal processes within the tube. Studies on mast cells and neuronal axons have reported characteristic sawtooth-shaped force fluctuations that emerge above the equilibrium plateau when tethers are held at constant extension (Farrell et al. 2013; Datar et al. 2015). In these works, the rises were attributed to F-actin polymerization, and the decays to depolymerization and active rearward transport by myosin II motors. A recent study revisited this behavior in tethers pulled from axons, combining OT force measurements with live imaging of actin and stochastic modeling (Ashraf et al. 2026). In these tethers, actin filaments were present within the tube and underwent cyclic growth and retraction, accompanied by repeated load-and-fail force events. These cycles required dynamic actin and ATP, but persisted after inhibition of myosin II and myosin V. ADF/cofilin colocalized with actin inside the tube, and a constitutively inactive ADF mutant reduced peak force and prolonged force events, supporting a mechanism in which ADF/cofilin-induced filament shrinkage, rather than motor activity, generates the contractile force (Ashraf et al. 2026). Twisting and coiling of the actin shaft within tether-like structures, driven by actomyosin activity, can also generate traction and buckling forces that contribute to the dynamic force signal (Leijnse et al. 2015, 2022). Helical buckling of actin inside tethers generated from axons has also been observed under conditions in which myosin II and myosin V activity were dispensable, and was interpreted as a signature of torsional stress generated by ADF/cofilin binding (Ashraf et al. 2026). In addition, OT-pulled tethers have been used as a controlled system to dissect the molecular machinery of actin polymerization within membrane tubes: inhibition of the Arp2/3 complex allows linear actin filaments to extend over greater distances inside the tube, a mechanism shared with TNTs (Henderson et al. 2023). Apart from F-actin itself, some studies also point, with limited spatial and temporal detail, to the presence of fascin, VASP, the I-BAR-domain protein IRSp53, and cofilin within tethers (Bornschlogl et al. 2013; Tsai et al. 2022; Ashraf et al. 2026), proteins more commonly associated with filopodia. How these and other proteins interact with F-actin inside a tether, and whether their presence changes its mechanical behavior, remain unclear.

These observations indicate that, at least in the cell types studied, a tether held under sustained force can be a mechanically active structure, shaped by ongoing cytoskeletal dynamics. What remains to be established, and constitutes an important open question for the field, is how these force dynamics evolve as a function of time under sustained localized force, how they relate to the state of the underlying cortex, and how the few molecular players identified so far fit into a comprehensive molecular map of the process. This is precisely the kind of question that OT-based approaches, combined with live fluorescence imaging of actin dynamics, are well positioned to address (Soares et al. 2022). However, the actin dynamics within the tether are only part of the story: sustained localized force also reorganizes the cortex at and beyond the site of application, driving structural changes that extend well past the nanotube itself.

### Cortical reorganization and protrusive structures

The idea that external mechanical force can reorganize the cell surface is well established, but most of what is known comes from experiments that apply forces globally, by stretching substrates, aspirating cells, or imposing fluid shear. What happens when force is applied locally, at a single point, is a different question, and one that is more directly relevant to the approach described in this review.

This difference matters. When a force is applied locally at a defined point on the cell surface, the cell stiffens and F-actin accumulates both at the contact site and up to several micrometers away, with the kinetics of stiffening and actin recruitment tightly correlated over minutes (Icard-Arcizet et al. 2008). AFM-based point loading confirms this local cortex stiffening (up to 1.35-fold in the vicinity of the loading site) and reveals that the extent of cytoskeletal reorganization scales with both the magnitude and duration of the applied force (Hu et al. 2019). This local response differs from global mechanical stimulation (Fig. 2). Repeated local force recruits actin and activates mechanosensitive transcriptional programs within 30 min, while global strain induces a distinct biphasic actin response and, at high intensities, paradoxically causes an intermediate phase of actin depolymerization (Montel et al. 2019). Both the nature and the magnitude of the mechanical stimulus determine which cytoskeletal structures are recruited, on what timescale, and with what downstream consequences.

This principle is not restricted to forces applied through cell–matrix adhesion receptors. Cells subjected to mechanical stress, whether by aspiration, substrate stretching, or localized probe contact, undergo cortical remodeling that involves both passive redistribution of membrane components and active cytoskeletal responses (Kosmalska et al. 2015; Sens and Plastino 2015). Membrane reservoirs are depleted and replenished in response to force, and the cortex adapts its thickness and organization to the mechanical state of the cell (Chugh and Paluch 2018). Furthermore, locally induced cortical remodeling has consequences that extend far beyond the site of force application. Rho-based cortical contraction induces cortical flow-based reorganization of membrane-cortex interactions that generates phosphoinositide signaling at the opposite side of the cell, demonstrating that local mechanical events at the cell surface are transduced into long-range gradients (De Belly et al. 2026).

Membrane geometry itself acts as an additional signal. Membrane extensions induced by mechanical aspiration or pharmacological treatments that weaken membrane-cortex coupling can give rise to lamellipodia-like protrusions, suggesting that the high-curvature geometry of a cylindrical membrane extension is itself sufficient to recruit curvature-sensing BAR domain proteins and bias the direction and organization of subsequent actin assembly (Tsai et al. 2022; Henderson et al. 2023). In addition, tether-like membrane extensions are known to template lamellipodia formation physiologically, for example during epithelial wound healing in vitro and in vivo (Korkmazhan et al. 2022; Kennard et al. 2023), raising the possibility that OT-pulled tethers could recapitulate a mechanism the cell already uses as part of its normal migratory repertoire. Whether OT-pulled tethers held under sustained force reproduce this actin nucleation and give rise to protrusions remains an open question in the field.

### Toward intercellular connections: a working hypothesis linking tethers, protrusions, and tunneling nanotubes

**Fig. 4:**
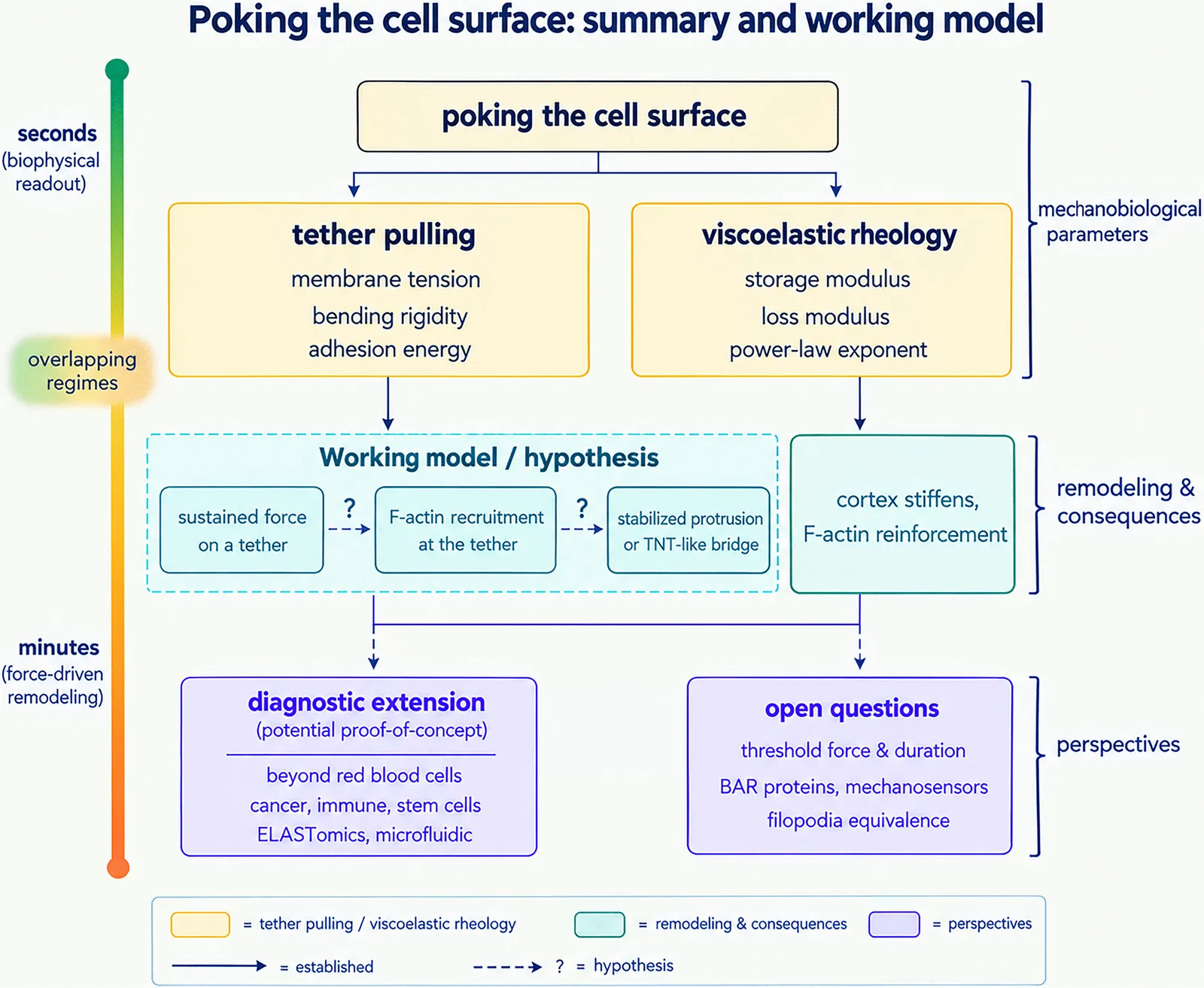
Poking the cell surface: summary and working model. Top (mechanobiological parameters): localized force applied to the cell surface by tether pulling or by oscillatory viscoelastic rheology yields the mechanobiological parameters described in “Force as a readout (seconds): biophysical parameters of the cell surface” section, namely membrane tension, bending rigidity and MCA energy from tether pulling, and storage modulus, loss modulus and power-law exponent from rheology. Middle (remodeling and consequences): sustained local stress stiffens the cortex and reinforces F-actin near the site of force application (solid box; “Cortical reorganization and protrusive structures” section). The dashed box marks a working model, proposed but not demonstrated: sustained force applied to a tether could progressively recruit F-actin, and such structures might evolve into stabilized protrusions or TNT-like bridges (“F-actin in tethers: evidence and controversy” and “Toward intercellular connections: a working hypothesis linking tethers, protrusions and tunneling nanotubes” sections); dashed arrows and question marks denote these untested steps. Bottom (perspectives): the two perspectives discussed in “Perspectives” section, namely the extension of mechanical readouts to diagnosis beyond RBCs, which remains at the proof-of-concept stage (“Biophysical diagnosis of cell surface pathology” section and Box 2), and the open questions about force and duration thresholds, the molecular players involved and the relationship of force-induced structures to filopodia and TNTs (“Open questions: force, actin, and the remodeling of the cell surface” section). The vertical gradient on the left indicates that the readout and remodeling regimes overlap in time. Box colors, solid arrows (established) and dashed arrows with question marks (hypothesis) are defined in the key at the bottom

Under the conditions in which F-actin has been detected (“F-actin in tethers: evidence and controversy” section), a tether held under sustained force is, mechanically, a membrane nanotube under tension that may contain an actin core (Pontes et al. 2011, 2013; Bornschlogl et al. 2013; Leijnse et al. 2015, 2022). To a first approximation, this description shares features with a TNT. It is therefore proposed here, as a working hypothesis rather than an established relationship, that force-extended membrane structures and intercellular nanotubes may share aspects of their formation mechanisms, molecular machinery and mechanical requirements. If this hypothesis is correct, tether extraction could provide a controlled experimental system for interrogating the structural transitions that produce TNTs physiologically (Fig. 4, working-model region). Direct mechanistic evidence for this link is currently lacking.

TNTs are thin, actin-containing membrane bridges that form between cells and support the transfer of organelles, vesicles, and signaling molecules over distances that exceed the length of canonical filopodia (Rustom et al. 2004; Zurzolo 2021). Their formation is incompletely understood, but two mechanisms were initially described: (i) outward growth from the cell surface driven by actin polymerization and (ii) cell dislodgement, in which a retraction fiber-like protrusion left behind by a migrating cell maintains membrane continuity with a neighboring cell and is subsequently stabilized into a TNT (Rustom et al. 2004; Onfelt et al. 2006; Ljubojevic et al. 2021). More recently, a third mechanism has been proposed (iii) in which TNTs form from double filopodial bridges through helical mechanical deformation (mechanical energy accumulated in a twisted bridge anchored by cadherin-cadherin interactions), suggesting that stored mechanical energy can drive the formation of close-ended TNT-like connections (Chang et al. 2022).

Mechanobiological studies have also begun to establish the quantitative framework for TNT mechanics. Using OT, the first measurements of TNT elastic properties demonstrated that these structures are force-bearing, can deform without rupture and exhibit bifurcations under applied force (Pontes et al. 2008). This capacity has been further confirmed by AFM, which quantified TNT stiffness and showed that external forces influence both their stability and cargo-transfer efficiency (Li et al. 2022). Notably, TNTs formed by actin-driven protrusion (HEK293 cells) and by contact and cell dislodgement (C2C12 cells) were reported to have similar tensile strength, which in both cases depended on tube length and diameter (Sun et al. 2024). A micropipette-based study has further shown that TNTs can transmit mechanical signals directly between connected cells, inducing calcium influx and altering the viscoelastic state of the recipient cell, revealing TNTs not merely as passive conduits but as mechanically active links in intercellular communication (Wang et al. 2024).

The cell-dislodgement mechanism is mechanically reminiscent of tether pulling: in both cases, a membrane tube forms under tension as two connected surfaces move apart, and the stability of the resulting tube depends on the balance between membrane tension, bending rigidity, and the presence of an actin core capable of resisting retraction. TNTs arising from these two distinct routes converge on comparable tensile properties (Sun et al. 2024), suggesting that the mechanical identity of such tubes may be determined more by their final architecture (a membrane tube under tension with an actin core) than by the pathway through which they formed. If so, a tether extended and held by an external force, which mimics the geometry of the dislodgement route, might in principle reach a comparable mechanical state. This remains a conjecture: no study has yet compared the mechanical properties of a force-extended tether with those of a physiologically formed TNT in the same cell type. Whether sustained localized forces at cell–cell contact sites contribute to TNT initiation or stabilization in vitro or in vivo and whether OT-based tether extension could serve as a controlled model for studying TNT mechanics are open questions that warrant direct investigation. The broader implication is that the cell surface, when poked with sustained localized force, may recapitulate aspects of the membrane dynamics that underlie intercellular communication. Understanding the mechanical requirements for transitioning from a force-extended tether to a stable intercellular bridge, in terms of force magnitude, duration, actin nucleation machinery, and membrane-cortex coupling, would significantly advance the understanding of how TNTs and related structures form and function. This is a frontier where the tools of single-cell biophysics and the biology of intercellular communication can converge (Fig. 4).

## Perspectives

### Biophysical diagnosis of cell surface pathology

The RBC studies described in the “Red blood cells: paradigmatic model for membrane mechanics and disease” section show that mechanical parameters of the cell surface are altered in several diseases, in some cases in ways that parallel functional impairment. A natural question is whether this principle extends beyond hematology, that is, whether the mechanical fingerprint of the cell surface could serve as a readout of pathological states in nucleated cells, where the membrane-cortex interface is richer, more dynamic, and more deeply embedded in signaling (Urbanska and Guck 2024). Proof-of-concept studies in several cell types and disease contexts suggest that it might (Fig. 4), but, as discussed below and in Box 2, the mechanical differences reported so far have not yet been translated into validated diagnostic tools.

The sensitivity of cell surface mechanics to molecular-level alterations in the membrane-cytoskeleton, demonstrated in RBC diseases, points toward a broader diagnostic potential. Mechanical measurements can detect changes in the membrane-cytoskeleton before overt morphological alterations are apparent, as recently shown for RBCs under chemical perturbation (Dorn et al. 2026), which suggests potential sensitivity to early pathological remodeling. For RBCs, however, the evidence summarized in this review for malaria, hereditary membrane disorders, sickle cell disease, β-thalassemia, and diabetes remains largely at the proof-of-concept stage.

Extending these approaches to nucleated cells, such as tumor cells, immune cells or stem cells, opens the possibility of biophysical phenotyping as a complement to molecular profiling. Cancer cells are generally reported to be mechanically softer than their normal counterparts, a difference detectable by AFM and OT-based measurements, and this softening has been correlated with invasive potential (Suresh 2007). AFM measurements can further distinguish between tumor subtypes (carcinoma and adenocarcinoma kidney cells show quantitatively distinct mechanical signatures despite both being softer than non-tumorigenic cells), suggesting that mechanical phenotyping may resolve tumor heterogeneity beyond the simple tumor-versus-normal comparison (Rebelo et al. 2013). Further evidence that viscoelastic parameters reflect functional cell state comes from systematic AFM measurements across cancer and non-cancer cell lines: the crossover timescale between fast and slow power-law relaxation regimes correlates with collective migration and invasive potential, providing a mechanical readout of malignancy that static stiffness measurements alone cannot offer (De Sousa et al. 2020). Immune cell activation is accompanied by changes in membrane tension and cortical stiffness that regulate phagocytosis (Masters et al. 2013) and cytotoxicity (Basu et al. 2016; Friedman et al. 2021). Stem cell differentiation involves transitions in membrane elastic properties that reflect changes in cytoskeletal organization (Soares et al. 2020; Bergert et al. 2021; De Belly et al. 2021). In renal epithelial cells, elevated copper availability, modeling the copper overload that may accompany kidney injury, increases tether force through reactive oxygen species (ROS)-dependent remodeling of the actin cytoskeleton in vitro (Pompeu et al. 2026), suggesting that metal and redox dyshomeostasis may also be reflected in cell surface mechanics. In each of these contexts, mechanobiological parameters of the cell surface carry information about cell state that is not easily accessible by purely molecular approaches. This is where mechanomics could contribute as a complementary diagnostic framework. Rather than asking which gene is mutated or which protein is overexpressed, mechanomics asks how the cell responds mechanically, and answers with quantitative, label-free parameters that are coupled to physical processes involved in disease. In principle, a diseased cell could display an altered mechanical signature detectable at the single-cell level; whether such signatures can support diagnosis, staging or monitoring remains to be established (Box 2).

The concept of viscoelastic phenotyping, which uses the full time-dependent mechanical response as a multi-parameter fingerprint of cell state, has been proposed and illustrated in RBCs. There, the three coupled relaxation timescales described in the “Red blood cells: paradigmatic model for membrane mechanics and disease” section, associated with the membrane, the membrane-spectrin skeleton, and the cytosol, together with cell stiffness, define a viscoelastic phenotype that the authors propose as a potential biomarker (Gironella-Torrent et al. 2024). This approach exemplifies what mechanomics could offer: a set of coupled timescales and moduli that together reflect the organizational state of the cell surface, rather than a single stiffness value.

A complementary approach that addresses both throughput and the link between mechanical and molecular phenotypes is ELASTomics, which uses nanopore electroporation as a proxy readout of cell surface tension in thousands of cells while profiling their transcriptome, enabling the joint mapping of mechanical and molecular cell states at a scale inaccessible to force-spectroscopy approaches (Shiomi et al. 2024). Applied to cancer cell lines, senescent cells, and differentiating hematopoietic progenitors, ELASTomics indicates that cell surface tension is, at least in part, a transcriptionally regulated property whose molecular determinants can be identified at single-cell scale.

Realizing this diagnostic potential requires overcoming the throughput limitations of single-cell OT measurements. Microfluidic integration, automated bead delivery and attachment, and multiplexed force platforms are active development areas that could bring OT-based surface mechanics measurements toward clinical applicability (Bustamante et al. 2021). Parallel development of simpler proxies for membrane tension may provide complementary routes to scalable mechanical phenotyping (Colom et al. 2018; Sitarska and Diz-Munoz 2020).

***Box 2**** From proof of concept to clinical use: requirements for mechanical biomarkers*. Most studies cited in the “Red blood cells: paradigmatic model for membrane mechanics and disease” and “Biophysical diagnosis of cell surface pathology” sections compare healthy and diseased cells in small, single-center datasets. Before cell surface mechanical parameters can be used for diagnosis, staging or monitoring, several requirements must be met. (i) Standardization and calibration: measurement protocols, calibration procedures (trap stiffness, cantilever spring constant, acoustic or magnetic force calibration) and analysis models must be harmonized, and inter-laboratory reproducibility must be demonstrated; cross-laboratory comparisons have shown how strongly results depend on technique and protocol (Wu et al. 2018). (ii) Biological and pre-analytical variability: donor-to-donor and cell-to-cell variability, cell age (particularly for RBCs), metabolic state, temperature, storage time, anticoagulant and buffer conditions must be characterized and controlled. (iii) Throughput and statistical power: single-bead techniques typically measure tens of cells per condition, whereas clinical use requires population-level sampling, which may call for high-throughput approaches such as AFS, MTC, microfluidic deformability assays or ELASTomics (Shiomi et al. 2024; Urbanska and Guck 2024). (iv) Reference ranges and diagnostic performance: healthy reference ranges, sensitivity, specificity and receiver operating characteristic analysis must be established with appropriate statistics. (v) Independent validation: performance must be confirmed in prospective, blinded and independent clinical cohorts, rather than inferred from cross-validation within a single dataset. Until these steps are taken, mechanical differences between healthy and diseased cells should be regarded as candidate biomarkers rather than validated diagnostic tests.

### Open questions: force, actin, and the remodeling of the cell surface

Whereas the diagnostic perspective above rests on parameters measured in the readout regime, the actin-dependent remodeling observed under sustained force remains mechanistically incompletely understood. Observations of F-actin within tethers and dynamic force fluctuations under sustained force application raise a set of questions that define an active research frontier. For example: does sustained localized force drive a progressive, time-dependent engagement of the cytoskeleton that fundamentally transforms the tether? If so, what force magnitude and duration are required to trigger this transition, and what molecular events determine the threshold? The tools to address these questions (OT-based tether pulling combined with live fluorescence imaging of actin dynamics at defined timepoints) are available, and the answers would clarify whether what has been observed in fragments across several studies represents a general, conserved mechanism of force-driven surface remodeling. Other open questions include how does sustained localized force activate actin nucleation at or near the tether base? Is this driven by mechanosensitive proteins responding to membrane tension, by curvature-sensing BAR-domain proteins recruited to the high-curvature region where the nanotube meets the flat cell surface, or by retrograde flow of cortical actin concentrating filament ends at the base? These mechanisms are not mutually exclusive, and super-resolution imaging of proteins within tethers would help tease apart their relative contributions.

Beyond the tether itself, and within the working hypothesis outlined in the “Toward intercellular connections: a working hypothesis linking tethers, protrusions, and tunneling nanotubes” section, important questions arise about how protrusions that might be stabilized by external force would relate to protrusions generated by canonical inside-out pathways. Would filopodia formed spontaneously be mechanically equivalent to protrusions emerging from OT-extended tethers under sustained force, if such protrusions form? Would they use the same molecular machinery, or would external force access a distinct nucleation program? Can the outside-in force pathway interact with inside-out signaling cascades? For example, can external force at a specific membrane location bias the direction of subsequent actin polymerization?

Answering these questions, individually and in combination (Fig. 4), is what could turn localized force from an observed correlate of cell surface remodeling into a mechanistically understood driver of it.

## Conclusions

What happens when you poke a living cell? When force is applied briefly, the cell surface reveals itself as a mechanically complex material whose biophysical parameters report on the mechanical state of the cell and are altered in several diseases. When localized force is sustained, it can engage the cytoskeleton and reorganize the surface locally, raising the possibility that force itself acts as an instructive signal that shapes cell surface architecture. These two regimes are best viewed as overlapping ends of a continuum rather than as separate states. Whether and how force acts as such a signal, what molecular machinery is involved, and how it relates to physiological processes of protrusion and cell–cell contact are among the open questions in the mechanobiology field. The tools to address them, including OT combined with live fluorescence imaging, super-resolution microscopy, and microfluidics, are increasingly accessible. The readouts that emerge from these measurements could form a structured mechanical fingerprint of cell state, whose value for diagnosis and disease monitoring will depend on standardization and validation in independent cohorts (Box 2). In the longer term, understanding how sustained force reshapes the cell surface may also enable its controlled manipulation. The cell surface, when poked, turns out to have a great deal to say. We are only beginning to listen.

## Data Availability

No datasets were generated or analysed during the current study.
